# Concentration-Dependent Effects of MXenes on Viability and Odontogenic Differentiation of Human Dental Pulp Stem Cells

**DOI:** 10.3390/dj14090607

**Published:** 2026-09-18

**Authors:** Mariam Mowafy, Nada Desouky, Marwa Samir, Alaa Omar, Rehab Abdelmonem

**Affiliations:** 1 Faculty of Biotechnology, Misr University for Science and Technology, Giza 12566, Egypt; mariam.mowafy@must.edu.eg; 2 Oral Biology Department, Faculty of Dentistry, Modern University for Technology and Information, Cairo 12055, Egypt; marwa.moussa@dentistry.cu.edu.eg; 3 Nanomedicine Research Unit, Delta University for Science and Technology, Gamasa 11152, Egypt; alaa.bokharist@deltauniv.edu.eg; 4 Industrial Pharmacy Department, Faculty of Pharmaceutical Sciences and Drug Manufacturing, Misr University for Science and Technology, Giza 12566, Egypt

**Keywords:** MXenes, dental pulp stem cells, odontogenic differentiation, DSPP, RUNX2, osteocalcin

## Abstract

**Background/Objectives:** MXenes (transition metal carbides, nitrides, and carbonitrides) are promising 2D nanomaterials for biomedical applications due to their high conductivity, mechanical strength, and hydrophilicity. This study investigated the concentration-dependent effects of Ti_3_C_2_T_x_ MXenes on the viability, mineralization, and odontogenic differentiation of human dental pulp stem cells (DPSCs). **Methods:** Ti_3_C_2_T_x_ MXenes were synthesized and characterized by Scanning Electron Microscope with Energy Dispersive X-ray (SEM/EDX), Dynamic Light Scattering (DLS), zeta potential, UV-Vis, and conductivity analysis. DPSCs were characterized by their mesenchymal stem cell phenotype (CD73^+^, CD90^+^, CD105^+^, CD34^−^, CD45^−^). Cells were exposed to 10, 50, and 100 µg/mL MXenes. Cell viability was assessed by (3-(4,5-Dimethylthiazol-2-yl)-2,5-diphenyltetrazolium bromide) MTT assay and apoptosis was evaluated using Annexin V-FITC staining. Mineralization was assessed by Alizarin Red S staining. Odontogenic differentiation was evaluated by qPCR for DSPP, RUNX2, and OCN. **Results:** Characterization confirmed successful MXene synthesis with a zeta potential of −22 mV and conductivity of 6110 S/cm. MXenes at 10 and 50 µg/mLmaintained viability above 100%, while 100 µg/mL reduced viability to 65.6% at day 14 (*p* < 0.01). Apoptosis rates at 10 and 50 µg/mL (5.1% and 6.8%) were not significantly different from the control (4.2%), whereas 100 µg/mL significantly increased apoptosis to 28.4% (*p* < 0.01). Mineralization was enhanced at 10 and 50 µg/mL (*p* < 0.05 and *p* < 0.01) but reduced at 100 µg/mL (*p* < 0.01). Gene expression of DSPP, RUNX2, and OCN was upregulated 2–3-fold at 10–50 µg/mL but suppressed at 100 µg/mL (*p* < 0.01). **Conclusions:** Ti_3_C_2_T_x_ MXenes exert concentration-dependent effects on DPSCs. Moderate concentrations (10–50 µg/mL) promote metabolic activity, mineralization, and odontogenic differentiation, while high concentrations (100 µg/mL) induce apoptosis and cytotoxicity. These findings support the therapeutic potential of MXenes in regenerative dentistry, provided that concentration thresholds are carefully controlled.

## 1. Introduction

Nanotechnology, the manipulation of matter at atomic and molecular scales, creates characteristics not found in bulk materials [1]. This engineering at the molecular scale has been central to technological advancement during the last twenty years, enabling advances with higher performance and precision in electronics, energy storage, and medicine [2]. In this regard, a new class of two-dimensional (2D) materials, collectively called MXenes (transition metal carbides, nitrides, and carbonitrides), has emerged as a class of extremely diverse systems. They are characterized by high electrical conductivity, mechanical strength, and hydrophilicity, which together enable versatility for applications in both industrial and biomedical fields [3,4,5].

MXenes have shown promise in biomedical applications, including biosensing, drug delivery, photothermal therapy, and regenerative tissue engineering [1,2,6,7,8,9,10]. Their large surface area and tunable surface chemistry allow for efficient protein adsorption and cellular interactions, making them attractive candidates for stem cell modulation and tissue regeneration [11,12,13]. In particular, the unique surface chemistry and mechanical strength of MXenes appear favorable for promoting mineralization and bone regeneration, supporting their use in dental pulp stem cell (DPSC) models to elucidate their mechanism of action and establish safe concentration ranges for clinical translation [11,12,13].

Despite the growing interest in MXenes for biomedical applications, their effects on human DPSCs and odontogenic differentiation remain largely unexplored. Specifically, the concentration-dependent bioactivity of MXenes on DPSC viability, mineralization, and odontogenic gene expression has not been systematically investigated. Furthermore, the optimal concentration range that balances efficacy and safety for dental regenerative applications has yet to be established. This study addresses these gaps by evaluating the in vitro effects of Ti_3_C_2_T_x_ MXenes on DPSCs.

Here, we explore the application of MXenes as promising materials for dental pulp stem cell-based regenerative applications, specifically for bone defect repair and dentin regeneration. This study has two primary aims: (1) to provide mechanistic insight into the interaction of MXenes with DPSCs in vitro; (2) to assess their cytotoxicity and biocompatibility profile to establish concentration ranges that support viability and odontogenic differentiation, thereby facilitating clinical translation. To the best of our knowledge, this is the first study to systematically evaluate the concentration-dependent effects of Ti_3_C_2_T_x_ MXenes on the odontogenic differentiation of human DPSCs, including the expression of key markers such as DSPP, RUNX2, and OCN. We hypothesize that the distinctive surface chemistry and flexural properties of MXenes mediate enhanced cellular attachment, growth, and mineralization, thereby accelerating repair.

## 2. Materials and Methods

### 2.1. Study Design

An in vitro controlled experiment was performed to investigate how varying MXene concentrations affect the behavior of dental pulp stem cells (DPSCs) under odontogenic induction conditions. The DPSCs used in this study were characterized by their mesenchymal stem cell phenotype, including positive expression of CD73, CD90, and CD105, and negative expression of hematopoietic markers CD34 and CD45, as previously confirmed by the Stem Cells Center, Ain Shams Dental College. Cells were exposed to three different MXene concentrations: 10 µg/mL (low dose), 50 µg/mL (moderate dose), and 100 µg/mL (high dose). This setup resulted in four groups: untreated control group, DPSCs exposed to 10 µg/mL MXene, DPSCs exposed to 50 µg/mL MXene, and DPSCs exposed to 100 µg/mL MXene. The study examined cell viability, mineralization, and expression of odontogenic markers. All experiments were performed with three independent biological replicates (n = 3), each consisting of three technical replicates. Data are presented as mean ± standard deviation (SD). Statistical analysis assessed how MXene concentration influenced DPSC responses.

The concentrations of 10, 50, and 100 µg/mL were selected based on the established thresholds by Jang and Lee, who demonstrated that Ti_3_C_2_ MXene concentrations below 20 μg/mL promoted osteogenic differentiation, concentrations exceeding 50 μg/mL induced significant cytotoxicity, and the 50 μg/mL level served as a transitional ‘slightly cytotoxic’ threshold [14]. Prior to cell exposure, MXene stock solutions were ultrasonically re-dispersed for 15 min to ensure uniform particle distribution. It is important to note that the particle size and zeta potential of the working solutions were not re-measured in the cell culture media before cell exposure. This limitation is acknowledged and addressed in the Section 4. To minimize particle settling, MXene suspensions were freshly prepared and gently vortexed before each addition to cell cultures. It is important to note that while the MTT assay provides a reliable measure of metabolic activity, it does not directly distinguish between enhanced proliferation and increased metabolic rate. Additionally, complete dispersion in the culture medium may not be maintained over prolonged incubation periods, and the actual cellular exposure dose may vary over time. These limitations are addressed in the Section 4.

### 2.2. Ethical Approval

This study was approved by the Scientific Research Ethics Committee of the Faculty of Dentistry, Al-Azhar University (Cairo Boys), under reference number 1451/5318. Extracted human third molars were obtained from orthodontic clinics as discarded tissue. The ethics committee waived the requirement for individual informed consent as all samples were de-identified and would otherwise have been discarded.

### 2.3. Materials

Titanium carbide (TiC, 99.5%, Cat# 12382), Aluminum (Al, 99.5%, Cat# 10555), and Titanium (Ti, 99.5%, Cat# 14062) powders were purchased from Alfa Aesar (Haverhill, MA, USA). Hydrofluoric acid (HF, 48%, Cat# 202846) and hydrochloric acid (HCl, 37%, Cat# 202920) were supplied from Acros Organics (Geel, Belgium). Deionized water (Cat# W4502) was used in all experiments. All chemicals were used directly without further purification.

Dental pulp stem cells (DPSCs) were obtained from the Stem Cells Center, Ain Shams Dental College (Cairo, Egypt). For cell culture, Dulbecco’s Modified Eagle Medium (DMEM, Cat# 11965092) was purchased from Gibco (Thermo Fisher Scientific, Waltham, MA, USA) and supplemented with fetal bovine serum (FBS, Cat# F2442, Sigma-Aldrich, St. Louis, MO, USA) and penicillin-streptomycin (Cat# 17-602E, Lonza, Basel, Switzerland). Routine subculture procedures employed trypsin-EDTA (Cat# 25200072, Gibco, Thermo Fisher Scientific, Waltham, MA, USA).

For enzymatic digestion of pulp tissue, collagenase type I (Cat# LS004196, Worthington Biochemical Corporation, Lakewood, NJ, USA) and dispase (Cat# D4693, Sigma-Aldrich, St. Louis, MO, USA) were used. Fixation of cells was performed with paraformaldehyde (Cat# P6148, Merck, Darmstadt, Germany). Phosphate-buffered saline (PBS, Cat# 10010023) was purchased from Gibco (Thermo Fisher Scientific, Waltham, MA, USA).

Cell viability was assessed using MTT reagent (Cat# M5655, Sigma-Aldrich, St. Louis, MO, USA). Mineralization assays employed Alizarin Red S (Cat# A5533, Sigma-Aldrich, St. Louis, MO, USA), with cetylpyridinium chloride (Cat# C0732, Sigma-Aldrich, St. Louis, MO, USA) used for dye quantification.

For molecular analyses, RNA extraction was carried out using TRIzol reagent (Cat# 15596026, Invitrogen, Thermo Fisher Scientific, Waltham, MA, USA). Quantitative real-time PCR was performed with SYBR Green Master Mix (Cat# 4367659, Applied Biosystems, Waltham, MA, USA), employing primers specific for dentin sialophosphoprotein (DSPP), Runt-related transcription factor 2 (RUNX2), osteocalcin (OCN), and glyceraldehyde-3-phosphate dehydrogenase (GAPDH) as the internal reference. All primers were synthesized by Integrated DNA Technologies, Inc. (Coralville, IA, USA) (Table 1).

### 2.4. Methodology

#### 2.4.1. Preparation of MXenes

**Figure 1:**
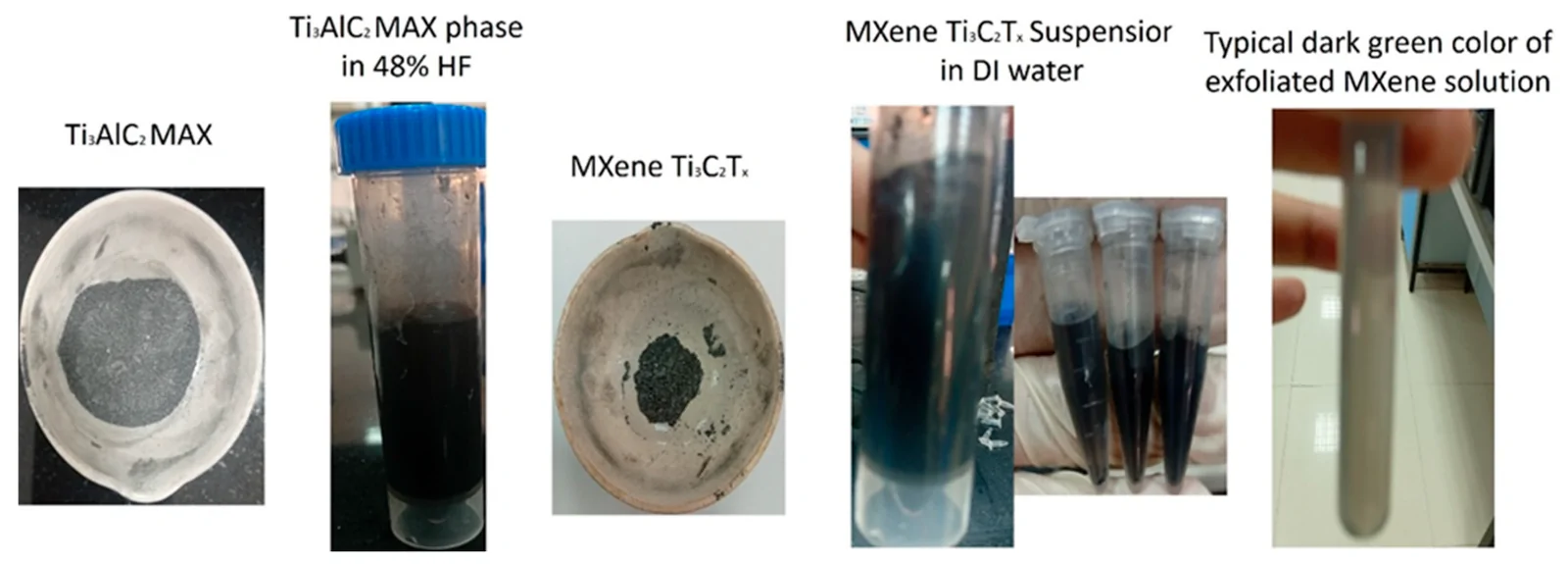
Schematic illustration of the Ti_3_AlC_2_ MAX phase precursor used for the synthesis of Ti_3_C_2_T_x_ MXene, showing the layered structure and the resulting few-layer dispersion of MXene at a concentration of 10 mg/mL.

Ti_3_AlC_2_ MAX phase was prepared by high-temperature annealing of TiC, Al, and Ti powders. An atomic ratio of 2:1:1 TiC:Ti:Al was used. The powders were mechanically activated via ball milling [15] at 60 rpm for 18 h with zirconia balls at a 2:1 ball-to-powder weight ratio. The milled powders were placed in a tube furnace with Ar flowing continuously through the tube at 200 cm^3^/min. The samples were heated to 1400 °C for 2 h at a heating and cooling rate of 3 °C/min. One gram of the Ti_3_AlC_2_ was gradually added over 5 min into a 60 mL polypropylene solution bottle or Teflon container containing 10 mL of a solution composed of 1 mL of 48 wt% HF, 3 mL deionized H_2_O, and 6 mL of 12 M HCl [16,17]. The reaction was allowed to continue for 18–24 h with stirring at 500 rpm at 35 °C. Once the reaction was complete, the mixture was washed with deionized water by centrifugation at 3500 rpm for 5 min to settle the powder and decant the clear supernatant. The washing cycles were repeated until the supernatant reached neutral pH (~6–7). Then, 14 mL of dimethyl sulfoxide (DMSO) was added to the material, and the reaction was continued under stirring at 240 rpm for 18 h. The DMSO was then washed several times with distilled water at 9000 rpm. The synthesized material was kept in a vacuum with activated silica to dry for 24 h. MXene (0.25 g) was collected as a powder, and the remaining amount was dispersed in a dimethyl sulfoxide and mixed water solution (Figure 1).

The prepared MXenes were then characterized using a scanning electron microscope (FEI, Hillsboro, OR, USA) with energy dispersive X-ray spectroscopy (SEM/EDX) to visualize the surface morphology and for elemental analysis, dynamic light scattering (DLS) to determine the size and distribution of MXene particles, ultraviolet-visible spectroscopy (UV-Vis) to confirm the optical absorbance profile of the material, and zeta potential analysis to assess the surface charge, which is related to colloidal stability.

For cell culture experiments, the MXene powder was re-dispersed in sterile culture medium and ultrasonicated for 15 min immediately before use to ensure uniform particle distribution. The hydrodynamic size and zeta potential of the working solutions were not re-measured prior to each experiment.

#### 2.4.2. Cell Isolation and Culture

Primary human dental pulp stem cells (DPSCs), previously isolated and characterized by the Stem Cells Center, Ain Shams Dental College (Cairo, Egypt), were used in this study. The cells were isolated from freshly extracted human third molars obtained under sterile conditions from orthodontic clinics. A total of three teeth from three independent donors were used, and the cells were pooled for all experiments. Immediately after extraction, teeth were rinsed with phosphate-buffered saline (PBS) to remove blood and debris, and the pulp tissue was carefully dissected from the crown and root chambers using sterile instruments. The pulp samples were enzymatically digested in a solution of collagenase type I (3 mg/mL) and dispase (4 mg/mL) at 37 °C for 1 h with gentle agitation to facilitate tissue breakdown. The resulting suspension was passed through a 70 µm cell strainer to obtain a single-cell suspension, which was then centrifuged at 300× *g* for 5 min. The cell pellet was resuspended in Dulbecco’s Modified Eagle Medium (DMEM) supplemented with 10% fetal bovine serum (FBS), 1% penicillin–streptomycin, and 2 mM L-glutamine. Cells were cultured at 37 °C in a humidified atmosphere containing 5% CO_2_, with medium changes every 2–3 days. Subculturing was performed at 70–80% confluence using 0.25% trypsin-EDTA, and all experiments were conducted using passage 3 cells to ensure consistency and minimize donor variability [18]. The mesenchymal stem cell phenotype of the cells (CD73^+^, CD90^+^, CD105^+^, CD34^−^, CD45^−^) was previously confirmed by the Stem Cells Center, Ain Shams Dental College.

### 2.5. Experimental Groups

Dental pulp stem cells (DPSCs) were divided into four experimental groups to evaluate the effects of MXenes on cell viability and behavior. Group 1 served as the untreated control, with DPSCs maintained in the odontogenic culture medium. Group 2 consisted of DPSCs exposed to MXenes at a concentration of 10 µg/mL, while Group 3 received MXenes at 50 µg/mL. Group 4 was treated with the highest concentration, 100 µg/mL MXenes. This grouping allowed for a systematic comparison between untreated cells and those exposed to increasing MXene concentrations, thereby enabling assessment of dose-dependent cellular responses.

### 2.6. Assessment Methods

#### 2.6.1. MTT Assay

**Figure 2:**
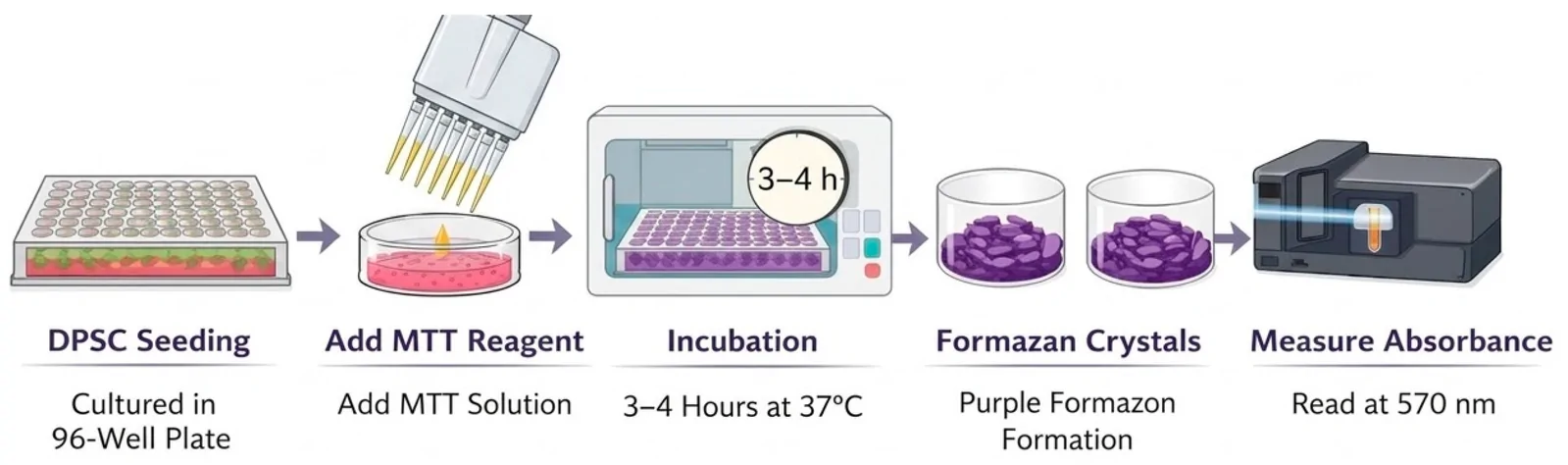
MTT assay results showing the viability of DPSCs after exposure to different concentrations of MXene (0, 10, 50, and 100 µg/mL) at days 1, 7, and 14. Data are presented as mean ± SD. Significant reductions in viability were observed at 100 µg/mL (*p* < 0.05, *p* < 0.01, *p* < 0.001).

Cell viability of dental pulp stem cells (DPSCs) exposed to different concentrations of MXenes was evaluated using the MTT assay. At days 1, 7 and 14, MTT solution (0.5 mg/mL) was added to each well and incubated for 4 h at 37 °C to allow the formation of formazan crystals. The crystals were dissolved in dimethyl sulfoxide (DMSO), and absorbance was measured at 570 nm using a microplate reader (Figure 2). Optical density values were normalized to vehicle controls, and cell viability was expressed as a percentage relative to the untreated control group [18].

Apoptosis was assessed using an Annexin V-FITC Apoptosis Detection Kit (BD Biosciences, Franklin Lakes, NJ, USA) according to the manufacturer’s instructions. Briefly, DPSCs were harvested after 14 days of treatment, washed twice with phosphate-buffered saline (PBS), and resuspended in 1× binding buffer at a concentration of 1 × 10^6^ cells/mL. Cells were then stained with Annexin V-FITC and propidium iodide (PI) for 15 min at room temperature in the dark. Apoptotic cells were quantified using a flow cytometer (BD FACSCalibur, BD Biosciences, Franklin Lakes, NJ, USA). Early apoptotic (Annexin V^+^/PI^−^) and late apoptotic (Annexin V^+^/PI^+^) cells were combined to calculate the total apoptosis rate. Data are expressed as mean ± SD from three independent experiments (n = 3).

#### 2.6.2. Alizarin Red Staining

Cells were initially rinsed with phosphate-buffered saline (PBS) following removal of the culture medium, then fixed using 3.7% paraformaldehyde prepared in PBS. After an additional PBS wash, mineral deposition was assessed by incubating the cells with a 1% (*w*/*v*) Alizarin Red S solution for 30 min. Excess dye was removed by washing with deionized water. For quantitative analysis, bound stain was released using a destaining solution consisting of 5% perchloric acid in deionized water for 30 min. Triplicate 100 μL aliquots from each well were transferred into a clear 96-well plate, and absorbance was measured at 405 nm using a microplate reader. Data were normalized against DNA content and vehicle-treated controls (0 μg/mL) [19].

#### 2.6.3. Quantitative RT-PCR

The extraction of total RNA from DPSCs on day 21 was performed using TRIzol reagent (Invitrogen, Thermo Fisher Scientific, Waltham, MA, USA) according to the instructions provided by the manufacturer. Briefly, DPSCs were lysed with TRIzol, and the RNA was isolated through phase separation with chloroform, followed by precipitation with isopropanol, washing with ethanol, and finally, the RNA was suspended in RNase-free water. The quality and concentration of RNA were determined spectrophotometrically. Samples were digested with DNase I to eliminate any genomic DNA contamination. Complementary DNA (cDNA) was reverse transcribed from total RNA, ranging from 500 ng to 1 µg, by employing random hexamer priming. Quantitative real-time PCR reactions were performed using SYBR Green Master Mix (Applied Biosystems, Waltham, MA, USA) and gene-specific primers for dentin sialophosphoprotein (DSPP), Runt-related transcription factor 2 (RUNX2), and osteocalcin (OCN); GAPDH was employed as an internal reference control (Table 1). Real-time PCR reactions underwent the following cycling steps: initial denaturation at 95 °C, followed by 40 cycles of 95 °C for 15 s and 60 °C for 30 s. The product specificity was confirmed by melt-curve analysis. Gene expression data were analyzed based on the ΔΔCt method and normalized to the endogenous control gene GAPDH and compared to the non-treated group [20].

### 2.7. Statistical Analysis

All data are presented as mean ± standard deviation (SD). Experiments were performed in triplicate. Statistical comparisons between groups were conducted using one-way analysis of variance (ANOVA) followed by Tukey’s post hoc test for multiple comparisons. Differences were considered statistically significant at a *p*-value < 0.05. All statistical analyses were performed using GraphPad Prism software (version 9.0).

## 3. Results

### 3.1. Characterization of MXenes

#### 3.1.1. SEM/EDX

**Figure 3:**
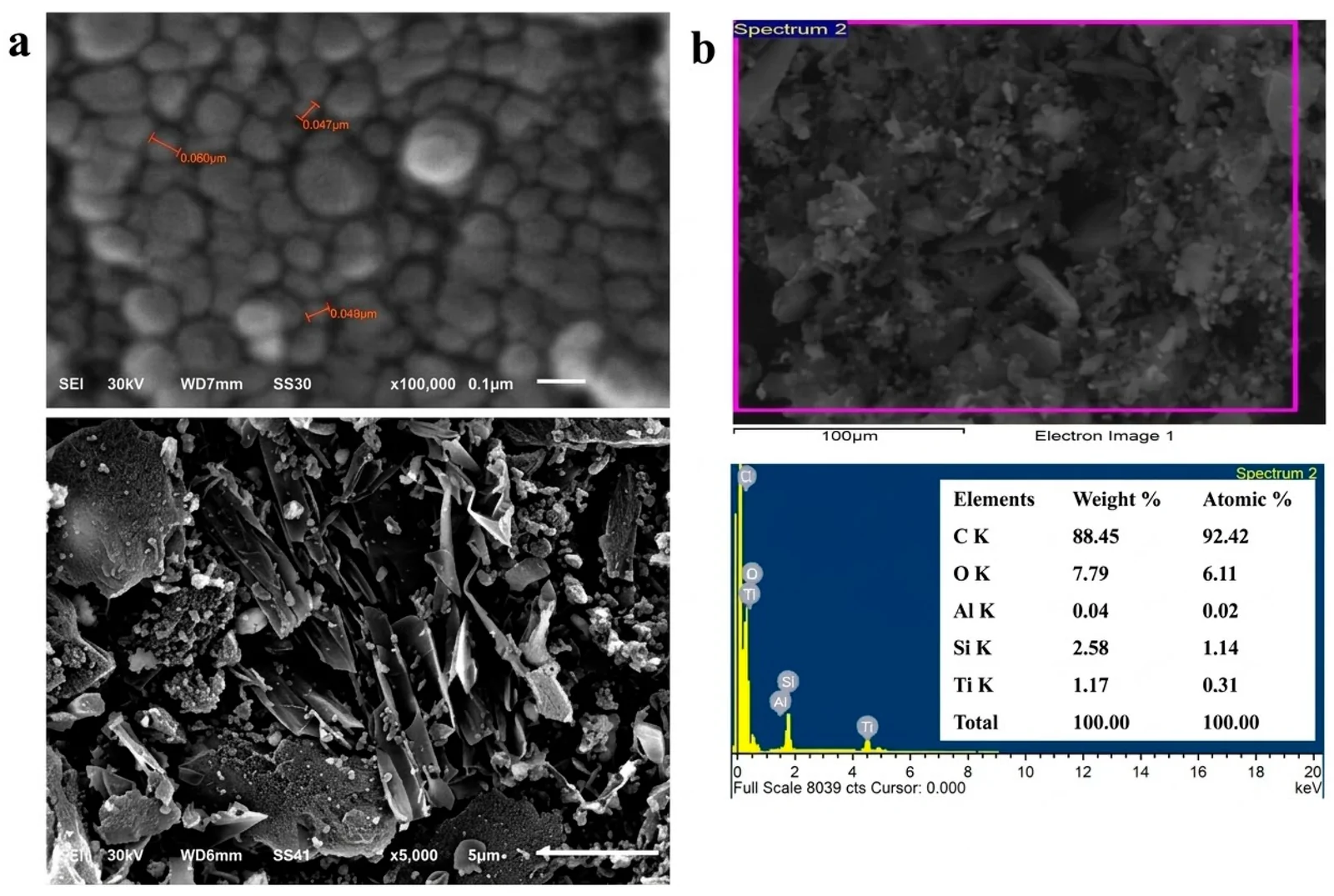
Characterization of Ti_3_C_2_T_x_ MXene. (**a**) SEM image showing the accordion-like layered morphology of the synthesized MXene nanosheets. (**b**) EDX spectrum confirming the presence of Ti, C, and O as the dominant elements, with minimal residual Al content (0.04 wt%, 0.02 at%), indicating successful etching of the MAX phase.

The 2D Ti_3_C_2_T_x_ microcomposite was examined by SEM. The SEM micrograph clearly shows the characteristic two-dimensional, few-layered MXene structure arranged in an accordion-like morphology (Figure 3a). The MXene sheets are tightly stacked, one atop another, as a result of van der Waals interactions and hydrogen bonding between surface terminations. SEM analysis revealed an average lateral dimension of 0.054 ± 0.008 µm (540 ± 8 nm).

To validate the chemical composition of the MXene Ti_3_C_2_T_x_ microcomposite, SEM-EDX analysis was conducted on the layered structure of the MXene (Figure 3b). The EDX spectra revealed the presence of Ti, C, and O as the dominant elements, which are characteristic of the main components of MXene. Additionally, a very low presence of Al was detected, with weight and atomic percentages of only 0.04% and 0.02%, respectively. This minimal Al content confirms the effective etching of the MAX phase and successful formation of the MXene. The elemental composition is consistent with previously reported values for Ti_3_C_2_T_x_ MXene, where titanium, carbon, and oxygen constitute the major components with trace amounts of aluminum from the MAX phase precursor.

#### 3.1.2. DLS and Zeta Potential

**Figure 4:**
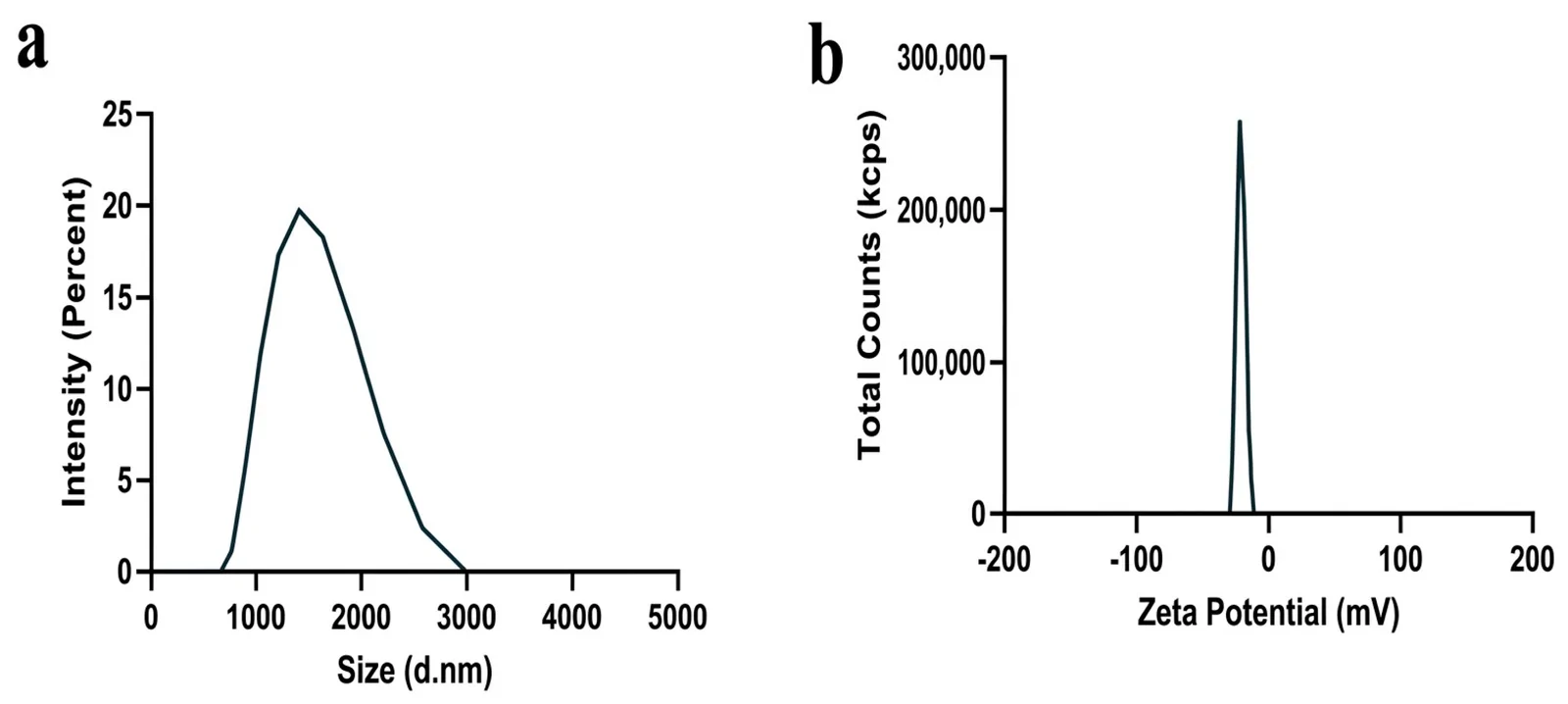
(**a**) DLS intensity−based size distribution of Ti_3_C_2_T_x_ MXene in aqueous solution, showing a mean hydrodynamic diameter of 1879.66 ± 228.45 nm. (**b**) Zeta potential distribution of Ti_3_C_2_T_x_ MXene, showing a peak value of −22 mV, indicating moderate colloidal stability.

The lateral dimension of the Ti_3_C_2_T_x_ microcomposite, measured by SEM, averaged 0.054 ± 0.008 µm (540 ± 8 nm), whereas dynamic light scattering (DLS) yielded a mean hydrodynamic diameter of 1879.66 ± 228.45 nm (Figure 4). The discrepancy likely arises because the ultrasonic treatment applied before SEM imaging further reduced the particle size relative to the static size measured by DLS. DLS is routinely employed to estimate the mean hydrodynamic size of nanomaterials such as Ti_3_C_2_T_x_ MXene and serves as an alternative to transmission electron microscopy (TEM) (Figure 4a). Zeta-potential measurements were also performed to assess the solution stability and surface charge of the MXene. DLS analysis revealed a peak zeta potential of −22 mV for the Ti_3_C_2_T_x_ microcomposite, indicating that the MXene dispersion is reasonably stable and that the MXene surfaces bear a negative charge (Figure 4b).

#### 3.1.3. UV-Vis

**Figure 5:**
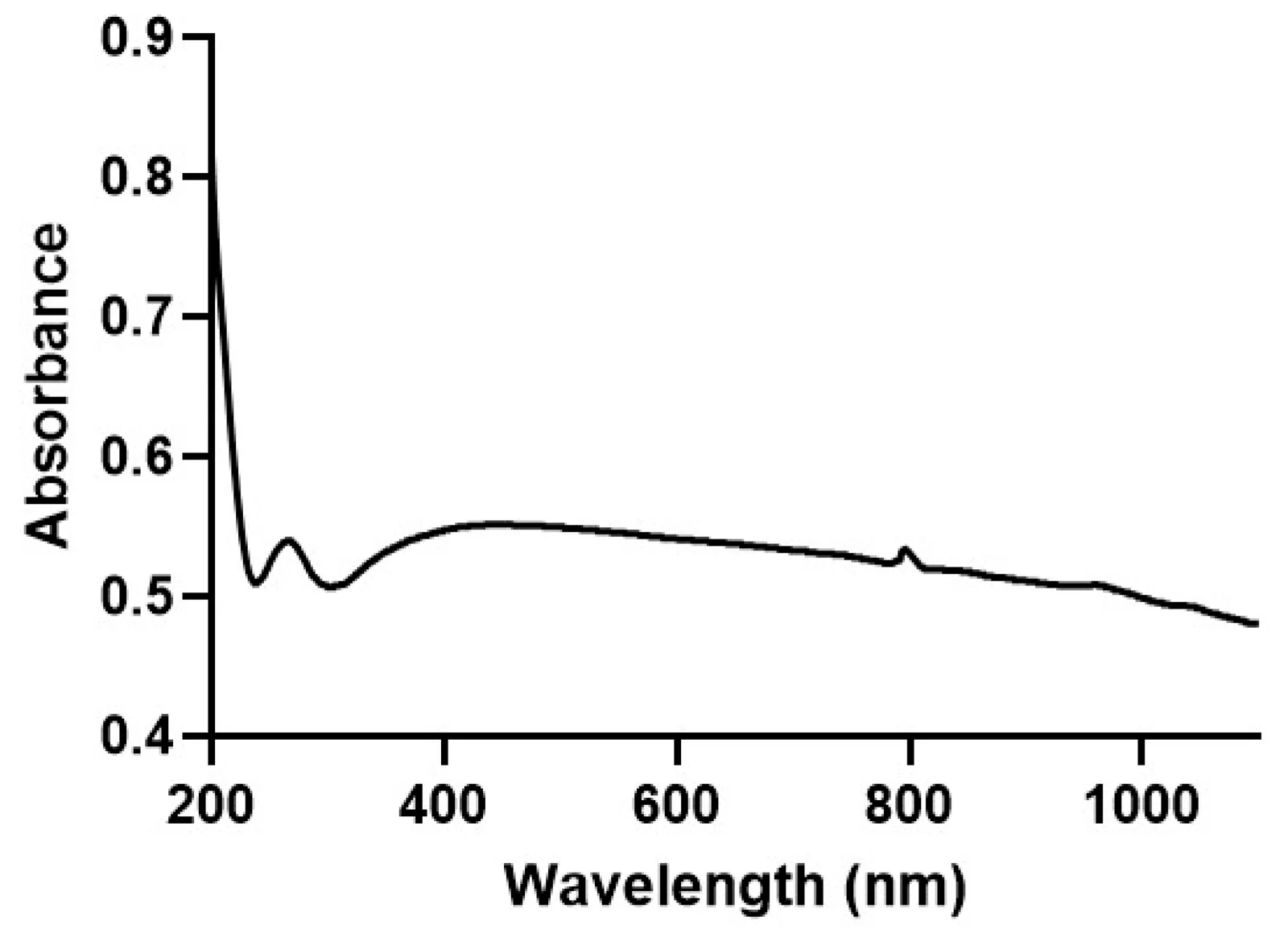
UV-Vis absorption spectrum of Ti_3_C_2_T_x_ MXene colloidal solution (1 mg mL^−1^) over the 200–1100 nm range. The spectrum shows a sharp absorption peak at approximately 350 nm, attributed to –OH and –O surface terminations, and a broad near-infrared (NIR) band centered at 795 nm, attributed to localized surface plasmon resonance.

For the investigation of the optical extinction of Ti_3_C_2_T_x_ dispersion, the UV–Visible spectroscopy method was employed in order to study both the absorption and scattering properties of the studied sample. Generally speaking, the optoelectronic properties of MXenes depend on the concentration of the MXenes dispersion, flake dimensions, and surface termination type. Figure 5 displays the absorption spectrum of the investigated MXene colloidal solution in the 200–1100 nm range. The Ti_3_C_2_T_x_ MXene dispersion (1 mg mL^−1^) was filtered using a 0.22 μm membrane before being subjected to analysis. The absorption spectrum shows high transmission in the visible light, significant absorbance at around 350 nm, which relates to –OH and –O surface terminations after etching, as well as a prominent absorption in the near-infrared region at 795 nm due to the localized surface plasmon resonance effect [21,22]. The analysis results also provide information about the concentration of the dispersion, according to the Lambert–Beer’s law A/l = Cα, where the absorption constant α = 29.2 mL mg^−1^ cm^−1^ [23], resulting in 0.10 mg mL^−1^ concentration. The conductivity of Ti_3_C_2_T_x_ MXene microcomposite was measured using a four-point probe method; the batch resulted with a conductivity of 6110 ± 150 S/cm.

### 3.2. MTT Analysis

The MTT assay revealed a concentration-dependent relationship between MXene concentration and DPSC viability. Cells exposed to 10 µg/mL MXene exhibited viability of 107.8 ± 3.1% at day 1, 106.8 ± 3.3% at day 7, and 106.7 ± 3.6% at day 14. Cells treated with 50 µg/mL MXene maintained viability levels of 105.5 ± 2.9%, 103.4 ± 2.7%, and 104.1 ± 3.0% at days 1, 7, and 14, respectively. In contrast, the 100 µg/mL group showed a marked reduction in viability to 84.9 ± 4.2% at day 1, 79.7 ± 4.5% at day 7, and 65.6 ± 5.1% at day 14 (*p* < 0.01).

To distinguish whether the reduced MTT viability at 100 µg/mL was due to cell death or metabolic inhibition, apoptosis was assessed using Annexin V-FITC staining. The control group exhibited a baseline apoptosis rate of 4.2 ± 1.1%. Treatment with 10 µg/mL and 50 µg/mL MXene resulted in apoptosis rates of 5.1 ± 1.3% and 6.8 ± 1.5%, respectively, which were not significantly different from the control (*p* > 0.05). In contrast, the 100 µg/mL group showed a marked increase in apoptosis to 28.4 ± 3.2% (*p* < 0.01), confirming that the cytotoxic effect at the highest concentration is associated with increased cell death rather than metabolic inhibition alone (Table 2).

### 3.3. Alizarin Red Analysis

**Figure 6:**
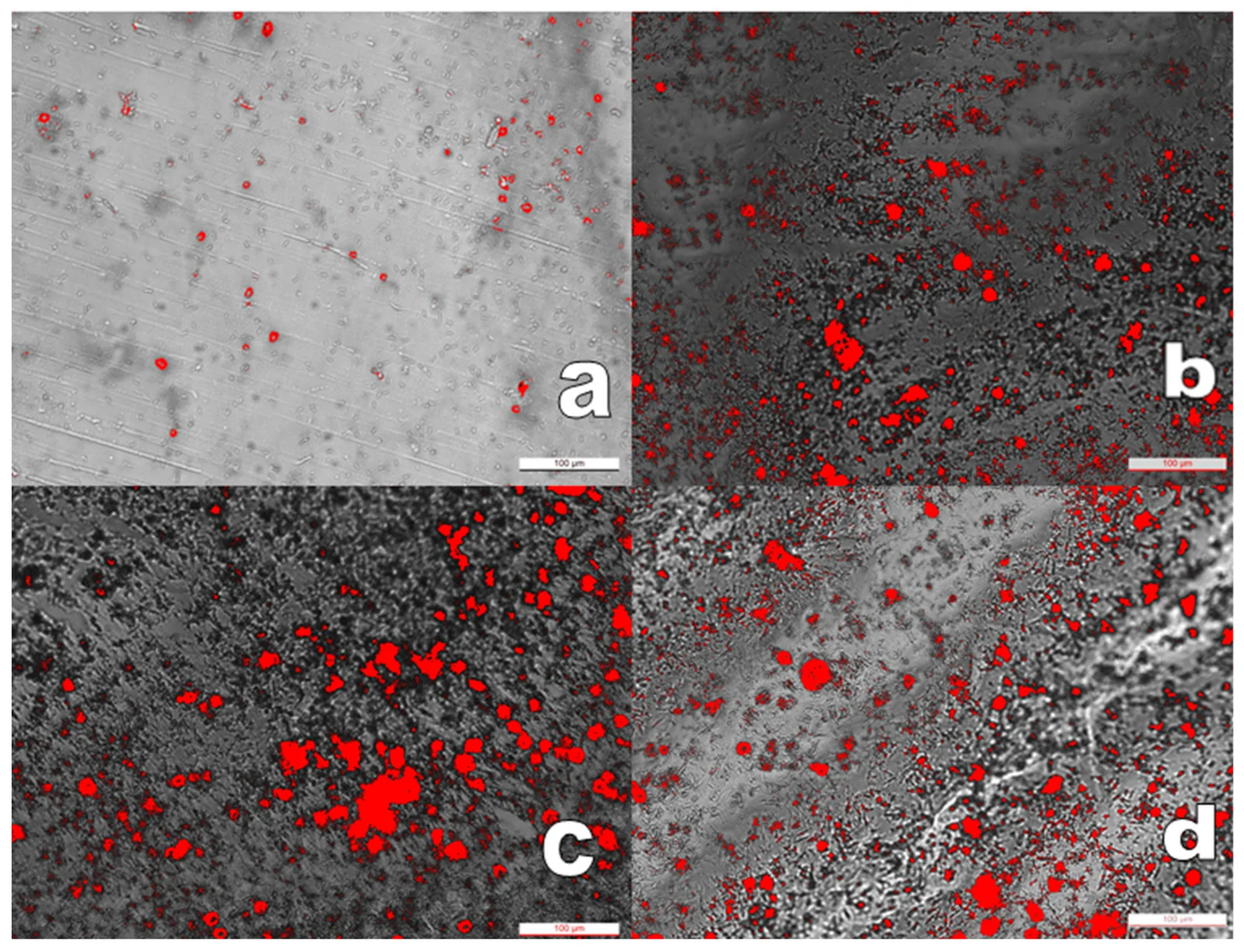
Alizarin Red S staining of DPSCs after 21 days of odontogenic induction. (**a**) Control group (0 µg/mL MXene) showing baseline calcium deposition. (**b**) 10 µg/mL MXene group showing increased red-stained mineralized nodules (absorbance: 0.48 ± 0.05, *p* < 0.05). (**c**) 50 µg/mL MXene group showing the most intense and extensive staining (absorbance: 0.62 ± 0.06, *p* < 0.01). (**d**) 100 µg/mL MXene group showing faint and sparse mineralization (absorbance: 0.15 ± 0.04, *p* < 0.01). Scale bar: 100 µm.

**Figure 7:**
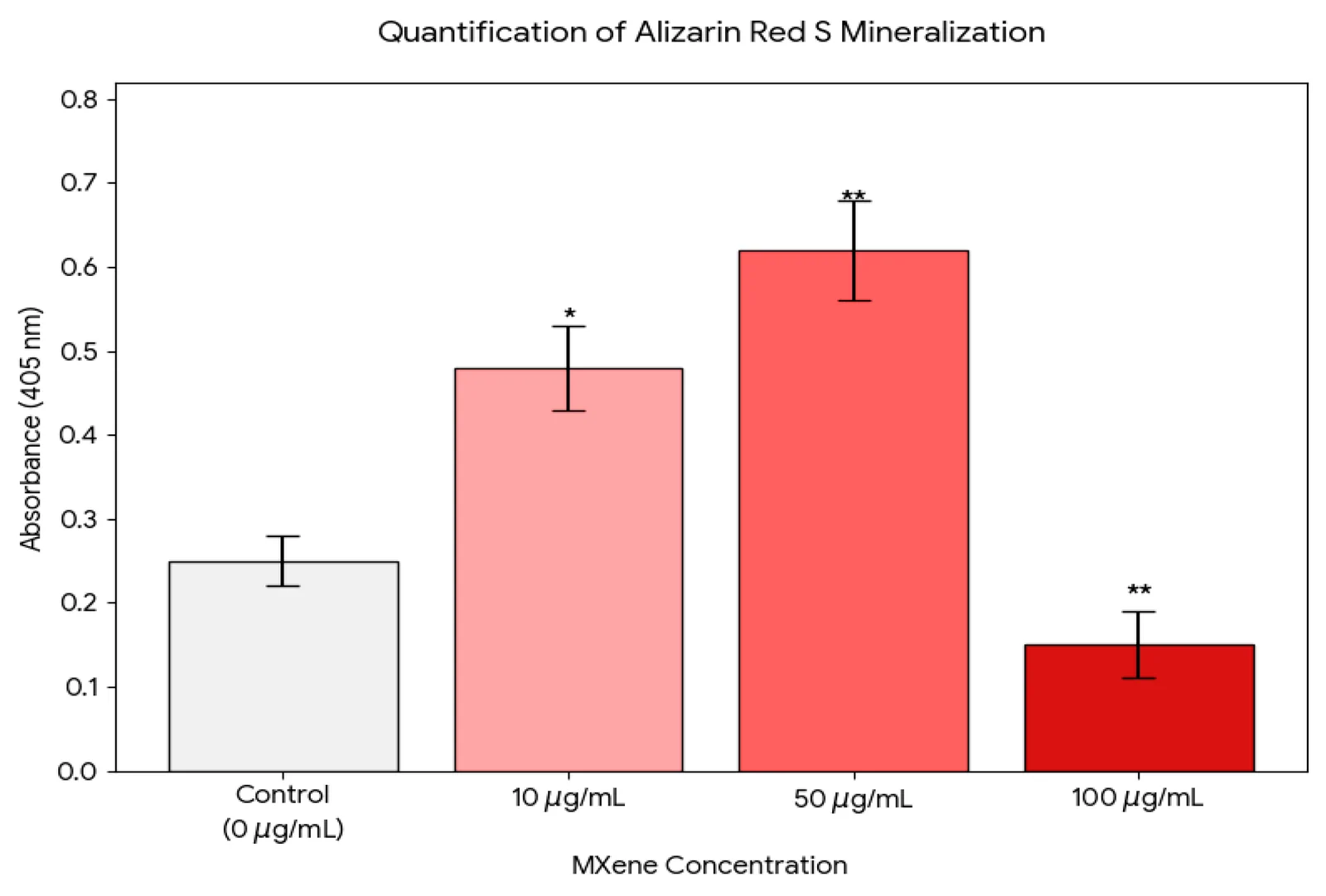
Quantitative assessment of calcium mineral deposition via Alizarin Red S (ARS) staining at 405 nm. Data are presented as mean ± SD. Significant increases in mineralization were observed at 10 µg/mL (*p* < 0.05) and 50 µg/mL (*p* < 0.01), while 100 µg/mL showed a significant reduction (*p* < 0.01) compared to the control. * *p* < 0.05, ** *p* < 0.01.

Assessment of the mineralization capacity of DPSCs in the presence of MXenes was performed using Alizarin Red S staining after 21 days of odontogenic induction. The absorbance results obtained from quantifying the samples revealed a significant dose-dependent effect. Treatment of DPSCs with MXenes at concentrations of 10 µg/mL and 50 µg/mL showed a significant increase in mineral deposition compared to the control group (*p* < 0.05). Among these, the 50 µg/mL concentration exhibited the highest absorbance values, indicating the greatest level of mineralization. In contrast, DPSCs treated with 100 µg/mL MXenes showed a significant decrease in staining (*p* < 0.01), indicating poor mineralization at this concentration. Although residual red staining was visible in Figure 6d, the quantitative absorbance was significantly reduced at 100 µg/mL (Table 3, Figure 7), suggesting that non-specific dye retention, rather than true mineralization, may account for this observation. This suppression correlates with the cytotoxicity observed in the MTT assay (Figure 6 and Figure 7; Table 3).

### 3.4. PCR Analysis

Gene expression profiling after 21 days demonstrated that MXenes significantly influence the odontogenic differentiation of dental pulp stem cells (DPSCs). At lower concentrations, MXenes facilitated differentiation, with DSPP expression increasing by 2.4-fold in the 10 µg/mL group (*p* < 0.05) and by 3.1-fold in the 50 µg/mL group (*p* < 0.01). Similarly, RUNX2 expression increased by 2.1-fold and 2.8-fold at 10 and 50 µg/mL, respectively, while OCN expression increased by 1.9-fold and 2.6-fold, respectively. These results reflect a strong stimulatory effect of MXenes on odontogenesis. In contrast, the highest concentration (100 µg/mL) led to marked reductions in gene expression: DSPP decreased to 0.45-fold, RUNX2 to 0.55-fold, and OCN to 0.60-fold relative to the control (*p* < 0.01 for all), indicating impaired differentiation. This decrease correlates with the cytotoxic effects and reduced mineralization observed at this concentration. Overall, these data demonstrate that MXenes promote odontogenic differentiation at non-toxic doses but inhibit it at toxic concentrations (Table 4).

## 4. Discussion

Recently, the investigation of 2D nanomaterials, specifically, MXenes, for biomedical purposes, including regenerative medicine, has gained prominence as a result of their unique physicochemical characteristics, namely high conductivity, hydrophilicity, and controllable surface functionality [24]. These materials’ ability to regulate the behavior of stem cells can make them effective options in regenerative dentistry, especially for DPSCs responsible for the regeneration of the dentin–pulp complex.

### 4.1. MXene Characterization

Scanning electron microscopy showed that the prepared Ti_3_C_2_T_x_ MXenes possessed the expected accordion-like layered morphology, whereas energy-dispersive X-ray spectroscopy confirmed that they consisted of Ti, C, and O elements with trace amounts of residual Al, confirming the efficient etching of the MAX phase. Dynamic light scattering suggested the hydrodynamic diameters were higher compared to the SEM lateral sizes due to the formation of hydration layers. The zeta potential was relatively low, meaning the material’s surface was charged through –OH and –O groups. The obtained ultraviolet–visible spectrum contained sharp absorption peaks in the ultraviolet region and broad near-infrared spectra attributed to plasmonic resonance. Conductivity tests confirmed high electrical properties of the examined material.

Similar to the present study, scanning electron microscopy (SEM) and energy-dispersive X-ray spectroscopy (EDX) have remained indispensable tools for MXenes characterization. Jothiramalingam et al. [25] have demonstrated that Ti_3_C_2_T_x_ nanosheets exhibit an accordion-like structure, characterized by layers of stacked nanomaterials. Further EDX analysis indicated that Ti, C, and O are major elements, whereas traces of aluminum were found. These findings validate previous studies and highlight the importance of morphology and purity of MXene elements.

Consistent with the current results, dynamic light scattering (DLS) has been widely used for analyzing MXene dispersions. According to Hekler et al. [26], the hydrodynamic diameters of Ti_3_C_2_T_x_ suspensions are significantly greater than lateral dimensions found through SEM analysis due to hydration layering and aggregation in aqueous medium. Thus, the DLS findings are well-supported by the literature and emphasize the significance of this technique.

UV–Vis spectroscopy has similarly been used to validate the optical properties of MXenes. For instance, Aliqab et al. [27] describe strong peaks of UV absorption and wide NIR bands linked to plasmon resonance, while Chaudhuri et al. [28] associate the peaks of UV absorption to –OH and –O terminations created through the etching process. Such findings correlate well with our results, suggesting that optical analysis plays an important role in assessing the quality of exfoliation and photothermal behavior.

Following a similar trend as ours, zeta potential analysis has been recognized as an effective test for assessing the stability of MXenes. For example, Natu et al. [29] reported a zeta potential value of close to −20 mV for Ti_3_C_2_T_x_ dispersion, indicating modest stability in aqueous solution and negative surface charges. Our study reveals similar results, indicating the importance of surface charges in determining dispersion stability.

Echoing our conductivity results, Zhang et al. [30] performed a study on MXene/sPS nanocomposites and observed increased electrical conductivity, whereas Hadi et al. [31] modeled tunneling conductivities of MXene composites. These findings confirm the electroactive properties of the Ti_3_C_2_T_x_ MXenes we have synthesized, which makes them appropriate for biosensors and regeneration scaffolds.

For cell culture applications, the MXene powder was re-dispersed in sterile culture medium and ultrasonicated for 15 min immediately before use to ensure uniform particle distribution. The hydrodynamic size and zeta potential of the working solutions were not re-measured prior to each experiment to confirm dispersion stability. However, we acknowledge that complete dispersion in the culture medium may not be maintained over prolonged incubation periods, and the actual cellular exposure dose may vary over time as particles may settle or aggregate. Additionally, the concentration of MXenes in the cell culture supernatant was not quantified over time, and therefore the exact dose reaching the cells remains uncertain. These limitations should be considered when interpreting the biological results.

### 4.2. Cell Viability and Apoptosis

The MTT assay revealed a concentration-dependent effect of MXenes on DPSC viability. Treatment with 10 and 50 µg/mL MXene maintained cell viability above 100% across all time points, suggesting that low to moderate concentrations may enhance metabolic activity. In contrast, 100 µg/mL significantly reduced viability to 65.6% at day 14, indicating a cytotoxic effect at higher concentrations. To further investigate whether this reduced viability was due to cell death or metabolic inhibition, apoptosis was assessed using Annexin V-FITC staining. The apoptosis rate at 100 µg/mL (28.4%) was significantly higher than the control (4.2%), confirming that the cytotoxic effect at this concentration is associated with increased cell death. In contrast, apoptosis rates at 10 and 50 µg/mL (5.1% and 6.8%, respectively) were not significantly different from the control, supporting the conclusion that these concentrations enhance metabolic activity without inducing cell death. These findings are consistent with Jang and Lee [14], who reported that Ti_3_C_2_ MXene concentrations below 20 µg/mL promoted osteogenic differentiation, while concentrations exceeding 50 µg/mL induced cytotoxicity. Collectively, these results demonstrate that MXenes exert a true concentration-dependent bioactivity on DPSCs, with moderate concentrations promoting metabolic activity without causing cell death, while high concentrations are cytotoxic.

### 4.3. Odontogenic Differentiation (DSPP, RUNX2, and OCN)

The results obtained from DSPP gene expression analysis support the positive influence of MXenes on odontogenesis at non-toxic concentrations, with 2.4-fold and 3.1-fold increases observed at 10 μg/mL and 50 μg/mL, respectively. This data is consistent with previous studies showing the presence of a stage-specific regulation of mineralization by DPSCs, DSPP being an important marker of odontogenesis [32,33].

Regarding RUNX2 and OCN expression, our findings demonstrate that MXene treatment at 10 µg/mL and 50 µg/mL significantly upregulated both RUNX2 (2.1-fold and 2.8-fold) and OCN (1.9-fold and 2.6-fold) expression. These results are consistent with another study which reported that Ti_3_C_2_ MXene particles promoted osteogenic differentiation of human mesenchymal stem cells with increased expression of RUNX2 and osteocalcin at concentrations below 20 μg/mL [14]. Similarly, it was demonstrated that surface-modified Ti_3_C_2_ MXene nanosheets promoted osteogenic differentiation of bone marrow mesenchymal stem cells via photothermal conversion under 808 nm NIR irradiation, with significant upregulation of RUNX2 and OCN [34]. Furthermore, It was demonstrated that a Ti_3_C_2_ MXene/nano-hydroxyapatite nanocomposite promoted osteogenic differentiation of BMSCs via photothermal conversion under 808 nm NIR irradiation, with enhanced osteogenic activity [35].

The 0.45-fold decrease in DSPP gene expression observed at 100 μg/mL suggests that cytotoxicity affects the odontogenic signaling pathway. Similarly, RUNX2 and OCN expression decreased to 0.55-fold and 0.60-fold, respectively, at the highest concentration, which is consistent with previous reports suggesting that high doses of MXenes may induce cellular stress and impair differentiation [36].

### 4.4. Proposed Mechanisms of Action

The stimulatory effects of MXenes at moderate concentrations may be attributed to their potential to modulate redox state and bioenergetics of cells, factors known to regulate stem cell differentiation [37]. Additionally, the surface characteristics of MXenes may facilitate protein adsorption and activation of odontogenic pathways such as BMP and Wnt, as previously suggested in the literature [33]. At higher concentrations, it is possible that MXenes induce cellular stress and mitochondrial dysfunction, which could lead to inhibition of stem cell differentiation and viability, as reported in other nanomaterial studies [36]. However, these pathways were not directly investigated in the present study.

Like bioactive cements and scaffolds, whose effect on enhancing the expression and mineralization of DSPP occurs at an optimal dose and becomes toxic at higher doses, MXenes show this dual nature depending upon the dose. This study presents new findings on how MXenes can directly influence the odontogenic differentiation of DPSCs, including the upregulation of RUNX2 and OCN, supporting their potential as bioactive materials for regenerative dentistry.

Several limitations should be acknowledged. The particle size and zeta potential of the MXene working solutions were not re-measured before cell exposure, and the actual concentration of MXenes reaching the cells over time was not quantified. Additionally, while the MTT assay measured metabolic activity, direct cell counting was not performed to confirm proliferation. Protein-level validation of odontogenic markers was not conducted, and the molecular mechanisms were not directly investigated. Finally, this was an in vitro study, and further animal studies are needed to support clinical translation.

## 5. Conclusions

This study demonstrates that MXenes exert concentration-dependent effects on the odontogenic differentiation of DPSCs. At moderate concentrations (10–50 µg/mL), MXenes significantly enhance mineral deposition and upregulate odontogenic markers including DSPP, RUNX2, and OCN, supporting their role as promising bioactive nanomaterials for dentin regeneration. However, at higher concentrations (100 µg/mL), MXenes induce cytotoxicity, suppressing both mineralization and odontogenic gene expression. These findings highlight the therapeutic potential of MXenes in regenerative dentistry, provided that concentration thresholds are carefully controlled.

## 6. Recommendations

MXenes show promise for regenerative dental applications when used within the 10–50 µg/mL concentration range, which balances efficacy with safety. The 50 µg/mL dose is particularly significant, as it produced the strongest DSPP expression and mineral deposition, making it a priority for animal model studies in bone defect repair and pulp capping. In contrast, 100 µg/mL caused marked cytotoxicity and impaired differentiation, underscoring the need for dose optimization and long-term biosafety evaluation before clinical translation.

Future research should also investigate surface functionalization with biomolecules or growth factors to enhance regenerative selectivity. Additionally, as MXenes possess a dark color, further studies are required to determine the optimal concentration that avoids undesirable color changes, particularly when used in restorative materials or in esthetically sensitive areas. This is essential to ensure patient acceptance and clinical applicability in visible regions of the oral cavity.

Finally, establishing standardized synthesis, sterilization, and characterization protocols for polymer–MXene nanocomposites is essential to ensure reproducibility, safety, and eventual clinical adoption.

## Figures and Tables

**Table 1:** Primers used in the study.

Gene	Forward Primer	Reverse Primer
DSPP	5′-CAACCATAGAGAAAGCAAACGCG-3′	5′-TTTCTGTTGCCACTGCTGGGAC-3′
RUNX2	5′-CCCAGCCACCTTTACCTACA-3′	5′-TTCCTGTCTGTGCCTTCTGG-3′
OCN	5′-CACTCCTCGCCCTATTGGCC-3′	5′-CCCTCCTGCTTGGACACAAA-3′
GAPDH	5′-AGCCACATCGCTCAGACAC-3′	5′-GCCCAATACGACCAAATCC-3′

**Table 2:** Long-term Cytotoxicity and Cell Viability.

Group	MXene Concentration	Day 1 Avg. Viability (%)	Day 7 Avg. Viability (%)	Day 14 Avg. Viability (%)	Apoptosis Rate (% of Cells)
Control	0 µg/mL	100.0 ± 2.1	100.0 ± 2.8	100.0 ± 2.5	4.2 ± 1.1
Low Dose	10 µg/mL	107.8 ± 3.1	106.8 ± 3.3	106.7 ± 3.6	5.1 ± 1.3
Medium Dose	50 µg/mL	105.5 ± 2.9	103.4 ± 2.7	104.1 ± 3.0	6.8 ± 1.5
High Dose	100 µg/mL	84.9 ± 4.2	79.7 ± 4.5	65.6 ± 5.1	28.4 ± 3.2

**Table 3:** Mineralization Potential (Alizarin Red S).

Group (MXene Concentration)	Absorbance (405 nm) Mean ± SD	*p*-Value (vs. Control)
Control (0 µg/mL)	0.25 ± 0.03	–
10 µg/mL	0.48 ± 0.05	<0.05
50 µg/mL	0.62 ± 0.06	<0.01
100 µg/mL	0.15 ± 0.04	<0.01

**Table 4:** Molecular Odontogenic Markers Expression.

Group (MXene Concentration)	DSPP Expression	RUNX2 Expression	OCN Expression
Control (0 µg/mL)	1.00 ± 0.12	1.00 ± 0.10	1.00 ± 0.11
10 µg/mL	2.40 ± 0.28 *	2.10 ± 0.25 *	1.90 ± 0.22 *
50 µg/mL	3.10 ± 0.35 **	2.80 ± 0.30 **	2.60 ± 0.28 **
100 µg/mL	0.45 ± 0.10 **	0.55 ± 0.12 **	0.60 ± 0.11 **

* *p* < 0.05, ** *p* < 0.01 compared to control.

## Data Availability

The raw data supporting the conclusions of this article will be made available by the authors on request.
